# The interplay between sexual activity, athletic performance, and recovery in athletes: a narrative review

**DOI:** 10.3389/fphys.2026.1797234

**Published:** 2026-06-17

**Authors:** Onur Akyüz, Mohammad Alimoradi, Mohammad Alghosi, Hüseyin Nasip Özaltaş, Caner Cengiz, Levent Ceylan, Yaşar Barut, Andreas Konrad

**Affiliations:** 1 Department of Coaching Education, School of Physical Education and Sports, Dicle University, Diyarbakir, Türkiye; 2 Department of Sports Injuries and Corrective Exercises, Faculty of Sports Sciences, Shahid Bahonar University of Kerman, Kerman, Iran; 3 HERC- Health, Exercise & Research Center, Dubai, United Arab Emirates; 4 Department of Physical Education, Technical and Vocational University (TVU), Tehran, Iran; 5 Ankara University, Ankara, Türkiye; 6 Faculty of Sport Sciences, Hitit University, Çorum, Türkiye; 7 Department of Child Development, Child Development Division, Faculty of Health Sciences, Ondokuz Mayis University, Samsun, Türkiye; 8 Institute of Human Movement Science, Sport and Health, Graz University, Graz, Austria

**Keywords:** athletic performance, autonomic nervous system, hormones, recovery of function, sexual behavior, sleep

## Abstract

Sexual activity is a common yet poorly understood aspect of athletes’ lives, frequently surrounded by cultural beliefs and traditional practices that lack consistent scientific support. Despite widespread assumptions that sexual activity impairs athletic performance or recovery, empirical evidence remains fragmented and often contradictory. This narrative review synthesizes current literature examining the physiological, psychological, and contextual effects of sexual activity on athletic performance and post-exercise recovery. Mechanistic pathways involving hormonal fluctuations, autonomic nervous system regulation, cardiovascular and neuromuscular responses, sleep quality, and psychological well-being are examined to clarify biologically plausible effects. Evidence indicates that sexual activity typically elicits mild physiological responses comparable to light physical exertion and does not meaningfully deplete energy stores or impair strength, endurance, power, or coordination when occurring several hours or more before competition. Acute hormonal changes, including transient variations in testosterone, cortisol, prolactin, and oxytocin, appear short-lived and unlikely to exert direct negative effects on performance. Psychological outcomes, such as reduced anxiety, improved mood, emotional bonding, and stress regulation, may support readiness and recovery in some athletes, while negative effects are more often linked to sleep disruption, interpersonal stress, or maladaptive timing rather than sexual activity itself. The review highlights that individual variability, belief systems, relationship context, sport-specific demands, and sleep timing play a larger role than physiology alone in shaping outcomes. A critical limitation of the current evidence base is the predominant focus on male athletes, which constrains sex-specific conclusions and clinical guidance. Current evidence does not support universal sexual abstinence before competition, though personalized strategies may be appropriate based on individual responses and preferences. Overall, sexual activity appears largely neutral with respect to athletic performance and may offer recovery-related benefits when appropriately timed and contextualized. Future research should prioritize controlled designs, sex-balanced samples, sport-specific analyses, and objective recovery markers to inform individualized, evidence-based recommendations for athletes and practitioners.

## Highlights

- The review synthesizes evidence showing that sexual activity several hours before competition is unlikely to deplete energy or negatively affect strength, endurance, power, or coordination. However, activity immediately before an event may cause temporary relaxation or fatigue, potentially affecting psychological readiness.
- The influence of sexual activity is heavily shaped by individual beliefs, relationship quality, cultural norms, and sleep timing. Psychological outcomes like reduced anxiety or improved mood can support recovery, while interpersonal stress, sleep disruption, or personal superstitions can have a stronger impact on performance than the physical act itself.
- The current evidence base is predominantly focused on male athletes, limiting sex-specific conclusions. Future research must prioritize female participants, account for menstrual cycle phases, and explore sport-specific effects to develop balanced, evidence-based, and individualized guidelines for all athletes.

## 1 Introduction

**Figure 1:**
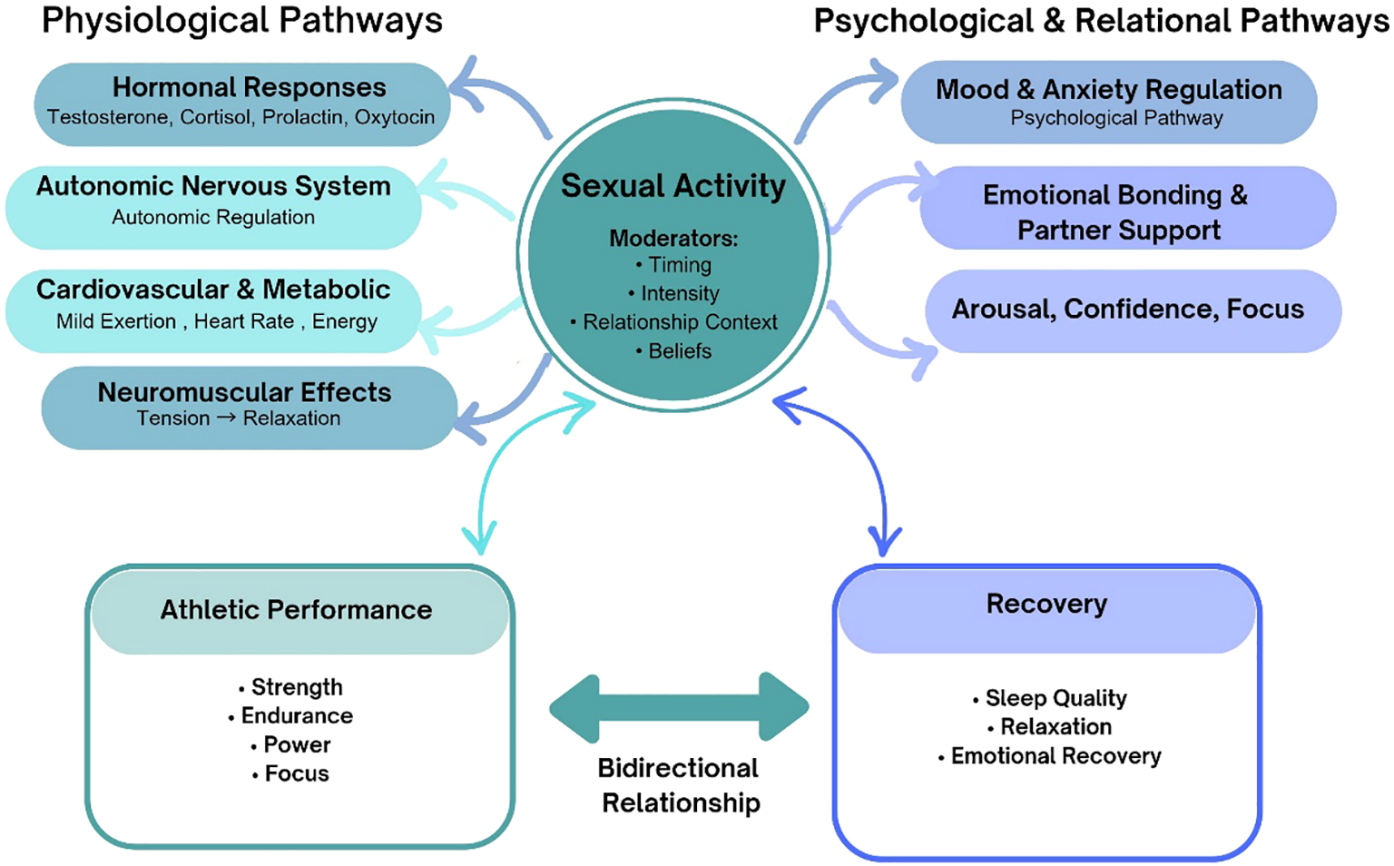
Physiological and psychological pathways linking sexual activity to athletic performance and recovery.

Athletic performance is influenced by an interplay of physiological, psychological, and social factors, and athletes routinely optimize training load, nutrition, sleep, and recovery to enhance adaptation and reduce injury risk (Gabbett, 2016; Alghurabi and Ravikanth, 2025; Mason et al., 2025). However, one commonly discussed but under-researched aspect of athlete life is sexual activity (Pappas, 2012). Across cultures and sporting traditions, beliefs persist that sexual activity may impair strength, aggression, focus, or recovery, despite limited and inconsistent scientific evidence (Anshel, 1981; Stefani et al., 2016; Soori et al., 2017). Biologically, sexual activity triggers a cascade of hormonal, autonomic, and neuromuscular responses that could influence acute performance and recovery (Soori et al., 2017; Zavorsky and Brooks, 2022). Hormones such as testosterone, prolactin, and cortisol fluctuate after sexual activity, autonomic balance shifts through changes in sympathetic and parasympathetic activity, and subjective states such as arousal, relaxation, and sleep quality may be altered in ways that are relevant to training or competition (Meston and Stanton, 2019; Lustig, 2021; Zavorsky and Brooks, 2022; Naragatti, 2025; Omona and Ssanyu, 2025). Psychologically and socially, sexual relationships contribute to emotional support, stress reduction, and mood regulation, all of which are known to affect motivation, adherence to training, and recovery after injury (Stefani et al., 2016; Fischer et al., 2022; Otadi, 2024). In contrast, concerns about fatigue, distraction, or disrupted sleep are frequently cited reasons for intentional sexual abstinence before competition (McGlone and Shrier, 2000). Despite these biologically and psychologically plausible pathways, scientific findings remain fragmented. Studies commonly use small samples, differing methodologies, and self-reported outcomes, and female athletes are significantly underrepresented (Zavorsky and Brooks, 2022). Cultural taboos and privacy concerns further limit open investigation (Zavorsky and Brooks, 2022). As a result, many athletes and practitioners rely on tradition, anecdote, or personal preference rather than evidence-based guidance (Stefani et al., 2016; Soori et al., 2017; Zavorsky and Brooks, 2022; Otadi, 2024). This gap leaves important questions unanswered, including whether sexual activity might have a negative effect on performance before competition, how timing influences endurance, strength, or skill-based performance, how effects differ between male and female athletes and among other athlete populations, and how sexual activity might influence recovery outcomes such as sleep, muscle soreness, or psychological resilience. This narrative review synthesizes current scientific evidence regarding the impact of sexual activity on athletic performance and recovery. It aims to clarify underlying physiological and psychological mechanisms, summarize existing empirical evidence, and highlight practical implications for athletes, coaches, and clinicians. Using a structured narrative approach, this review examines mechanistic pathways, pre-competition and training-period effects, post-exercise recovery factors, sex and sport-specific considerations, and key gaps in the literature. **Figure 1** provides a visual overview of the physiological and psychological pathways linking sexual activity to athletic performance and recovery, which are examined in detail in the following sections. The goal is to provide a balanced and evidence-informed foundation that supports individualized and practical decision making while guiding future research.

### 1.1 Search strategy

The foundation of this review is a comprehensive exploration of scientific literature. The authors conducted a structured literature search in PubMed, Scopus, and Web of Science from inception to December 2025. The following search string was used combining MeSH terms and keywords: (“sexual activity” OR “sexual intercourse” OR “sexual behavior” OR “coitus”) AND (“athletic performance” OR “sports performance” OR “exercise performance” OR “recovery” OR “sleep” OR “fatigue”) AND (“athletes” OR “sportspersons” OR “team sports” OR “endurance” OR “strength”). Additional hand searches of reference lists from relevant articles and previous systematic reviews were performed. Inclusion criteria were: original research, case studies, and peer-reviewed reviews examining sexual activity in relation to athletic performance or recovery in athlete or physically active populations; articles published in English. Exclusion criteria were: non-peer-reviewed sources (e.g., conference abstracts, editorials, opinion pieces), studies focusing exclusively on non-athlete populations, and articles where sexual activity was not the primary exposure variable.

## 2 Effects of sexual activity on athletic performance

The relationship between sexual activity and athletic performance has long been debated in sport science and practice. Traditional beliefs often suggest that sexual activity may impair strength, endurance, aggression, or concentration before competition, leading some athletes to practice pre-event abstinence. However, current evidence does not consistently support these assumptions (Stefani et al., 2016; Soori et al., 2017; Zavorsky and Brooks, 2022). Overall, research indicates that the effects of sexual activity on performance are minimal when it occurs several hours before exercise or competition. Observed outcomes are influenced more by timing, psychological state, and individual variability than by direct physiological disruption.

### 2.1 Sexual activity before competition

Sexual activity that occurs shortly before competition is the most widely investigated and controversial aspect of this topic. Studies examining these short-term effects show mixed findings (Stefani et al., 2016; Soori et al., 2017; Zavorsky and Brooks, 2022). Most research suggests that sexual activity completed several hours before competition does not impair physical performance in athletes (**Table 1**). Research examining handgrip strength, maximal voluntary contraction, power tests, and explosive movements typically find no reduction in performance when sexual activity occurs up to 24 hours before testing (Valenti et al., 2018; Zavorsky and Brooks, 2022). However, some athletes subjectively report reduced aggression or competitive drive if sexual activity occurs immediately before competition, which may influence sports that rely heavily on psychological arousal (Vajda, 2016). The mild cardiovascular load associated with typical sexual activity appears insufficient to deplete energy stores or alter aerobic performance (Zavorsky et al., 2019). Reaction time showed little measurable impairment following sexual activity, suggesting that sports requiring precision, accuracy, or tactical decision making are unlikely to be affected (Zavorsky et al., 2019). Although sexual activity up to 24 hours before competition appears neutral for most athletes, activity that occurs closer to performance—within hours of the event—may produce temporary fatigue, reduced alertness, or increased relaxation (Soori et al., 2017; Zavorsky and Brooks, 2022). The timing factor is therefore central when evaluating potential effects.

**Table 1:** Effects of sexual activity on athletic performance.

Study	Population	Timing before test	Wash-out period	Key findings	Performance impact
Valenti et al. (Valenti et al., 2018)	12 healthy physically active males (age: 25.6 ± 3.8 yrs)	12 hrs	3–7 days	Sexual activity was not detrimental to muscular strength.	Neutral
Vajda (Vajda, 2016)	67 combat sport athletes (age:18–61 yrs)	NR	NR	47% of athletes felt some change in sports performance connected to sexual activity; 83% perceived changes in the psychological component of performance. Physical effects were often perceived as negative, while psychological effects were often perceived as positive.	Mixed
Zavorsky et al. (Zavorsky et al., 2019)	7 male, 1 female (age 28 ± 5 yrs)	NR	7 days	No significant differences in physical work capacity, vertical jump height, handgrip strength, reaction time, or number of push-ups	Neutral
Johnson (Johnson, 1968)	14 married male, former athletes (age: 24–49 yrs)	8–12 hrs	6 days	Sexual intercourse the night before did not significantly affect strength or endurance the next morning.	Neutral
Anderson et al. (Anderson et al., 2001)	61 male and 14 female runners (age: 41.4 ± 10 yrs)	48 hrs	NR	Sexual activity was not related to athletes relative running performance.	Neutral
Vouyoukas (Vouyoukas, 2011)	7 male and 1 female participants (age: 27.9 yrs)	8–12 hrs	2 days	Sexual activity the night before did not impair aerobic capacity, vertical jump, grip strength, reaction time, hamstring flexibility and push-ups.	Neutral

NR, not reported.

### 2.2 Sexual activity during training periods

Regular sexual activity during training cycles may influence performance differently from single pre-competition encounters (Sgrò and Di Luigi, 2017). Chronic hormonal shifts, stress levels, sleep quality, and relationship satisfaction can affect how athletes respond to training loads (Chennaoui et al., 2016; O’Donnell et al., 2018). Evidence showed no detrimental effects of regular sexual activity on daily training (Stefani et al., 2016). In some cases, improved mood and emotional stability may enhance motivation and training adherence (Xu et al., 2025). However, poorly sexual activity may impair sleep, which can negatively affect subsequent training sessions (Pallesen et al., 2020; Charest and Grandner, 2022). During periods of heavy training, fatigue may be amplified by insufficient sleep or heightened psychological stress (Purvis et al., 2010; Surała et al., 2023). If sexual activity disrupts sleep or recovery routines, performance may decline (Pallesen et al., 2020; Charest and Grandner, 2022). Conversely, during lighter training phases or recovery days, sexual activity may support relaxation and emotional well-being without negative physical consequences (Levin, 2007; Zavorsky et al., 2019). Athletes vary widely in how arousal, relaxation, and energy levels influence training (Perkins et al., 2001). Some report improved readiness due to reduced anxiety, while others prefer abstinence due to personal beliefs or psychological preference (Sgrò and Di Luigi, 2017; Bondarenko and Demchenko, 2024). These subjective factors are important when interpreting scientific findings.

### 2.3 The sexual abstinence hypothesis in sport

The belief that abstinence enhances athletic performance has deep cultural and historical roots (McGlone and Shrier, 2000). Many coaches and athletes advocate for avoiding sexual activity in the days leading up to competition (McGlone and Shrier, 2000). Despite this tradition, scientific evidence provides limited support for the idea that abstinence improves physical performance (McGlone and Shrier, 2000; Stefani et al., 2016; Soori et al., 2017; Zavorsky and Brooks, 2022). The abstinence hypothesis is often based on assumptions that sexual activity reduces testosterone, drains energy, or decreases aggression (Dabbs and Mohammed, 1992; Marashi, 2024). Modern research does not consistently support these beliefs (McGlone and Shrier, 2000; Seftel et al., 2004). Testosterone levels may rise during arousal, and the energy expenditure of sexual activity is relatively low (van Anders et al., 2007; Oliva-Lozano et al., 2022). The psychological effects of abstinence may vary among athletes and may contribute to placebo or expectation-driven outcomes (Vajda, 2016; Vajda and Reguli, 2018). Beliefs about sexual behavior can significantly influence an athlete’s perceived readiness (Miller et al., 1998). Athletes who strongly believe abstinence improves performance may experience increased confidence and aggression when they abstain. Conversely, those who believe sexual activity helps them relax may perform worse if forced into abstinence. These psychological effects can be stronger than any physiological change (Alonso Aubin et al., 2023). Sports requiring high aggression or psychological arousal may be more sensitive to the psychological effects of sexual activity or abstinence (Alonso Aubin et al., 2023). In contrast, sports dependent on endurance, strategy, or fine motor control appears less affected (Anderson et al., 2001; Zavorsky and Brooks, 2022). Overall, empirical evidence does not broadly support the abstinence hypothesis, although individual preferences may justify personalized approaches.

Across available studies, sexual activity does not consistently impair performance when performed at least several hours before competition. Effects are more likely to be psychological than physiological, with individual beliefs, cultural background, and personal comfort playing a significant role. The strongest determinant of performance appears to be timing, particularly regarding sleep quality and mental readiness.

## 3 Sexual activity and recovery

Recovery is a multifaceted process that includes physiological restoration, psychological relaxation, hormonal regulation, and sleep quality (Desai et al., 2024; Kellmann et al., 2024; Otadi, 2024). Because sexual activity engages both bodily and emotional systems, it has the potential to influence several aspects of recovery after training or competition (Otadi, 2024). While research on this topic is limited, existing evidence suggests that sexual activity can positively or negatively affect recovery depending on timing, context, and individual responses. This section examines physiological restoration, sleep quality, psychological recovery, and potential risks.

### 3.1 Physiological recovery after exercise

Sexual activity may influence short-term physiological recovery through autonomic and hormonal changes. Following orgasm, there is a shift toward increased parasympathetic activity, accompanied by reductions in heart rate and perceived physiological arousal (Xue-Rui et al., 2008; Wang et al., 2010). This state is consistent with relaxation responses observed in other recovery strategies such as breathing exercises or meditation (Wu and Lo, 2008; Wang et al., 2010). Hormonal responses, including transient increases in prolactin and oxytocin and reductions in cortisol under relaxed conditions, may further contribute to a state of calm and reduced stress (Brody and Krüger, 2006; Hamilton et al., 2008). These changes suggest a potential role in facilitating post-exercise downregulation of physiological stress. However, there is currently no direct evidence that sexual activity improves muscle repair, reduces inflammation, or accelerates recovery of muscle damage. Any potential benefits are therefore likely indirect and mediated through stress reduction and improved relaxation rather than direct tissue-level effects. Given the low energy cost of sexual activity, it is also unlikely to meaningfully interfere with glycogen restoration or metabolic recovery (Holden‐lund, 1988; Frappier et al., 2013; Lew-Starowicz, 2022).

### 3.2 Sleep quality and restoration

Sleep is a central component of recovery, and sexual activity may influence sleep through both physiological and psychological pathways (Pallesen et al., 2020; Alghosi et al., 2025). Post-orgasmic increases in prolactin and oxytocin, combined with reduced sympathetic arousal, are associated with relaxation and may facilitate sleep onset in some individuals (Lastella et al., 2019). Improved sleep initiation and perceived sleep quality may indirectly support recovery processes such as hormonal regulation, immune function, and tissue repair (Kaczmarek et al., 2025; Langlais et al., 2025). However, these effects are highly dependent on timing. Sexual activity close to habitual bedtime may delay sleep onset or reduce total sleep duration in individuals with fixed morning schedules, thereby counteracting potential benefits (Chaput et al., 2020). Thus, the impact of sexual activity on sleep is bidirectional: it may support relaxation when well-timed, but impair recovery if it delays sleep or disrupts sleep hygiene routines.

### 3.3 Psychological recovery and emotional well-being

Recovery encompasses not only physical restoration but also psychological resilience and emotional balance (Wells, 2014). Sexual activity can play a significant role in this domain due to its effects on bonding, mood, and stress management (Burleson et al., 2007). Sexual activity is associated with increased release of endorphins and oxytocin, both of which elevate mood and promote feelings of closeness and relaxation (Olff et al., 2013). These effects may help athletes cope with the stress of competition, high training loads, or the emotional demands of travel and scheduling. Healthy and supportive sexual relationships may contribute to emotional stability, improved stress coping, and better adherence to recovery routines (Boucher et al., 2016). Emotional intimacy can also reduce anxiety or tension that might interfere with recovery (Boucher et al., 2016). In contrast, sexual activity associated with conflict, guilt, pressure, or interpersonal tension may negatively affect mood and recovery (Soller et al., 2017). Athletes who feel conflicted about sexual activity due to cultural beliefs or coaching restrictions may experience psychological stress that interferes with regeneration (Anshel, 1981; Maïn-Ndeiang et al., 2025).

### 3.4 Sexual activity during injury or rehabilitation

Athletes undergoing rehabilitation may experience physical limitations, discomfort, or emotional distress that influence sexual activity (Otadi, 2024). Conversely, healthy sexual relationships may support emotional resilience and reduce feelings of isolation during recovery from injury (Otadi, 2024). Some individuals report temporary reductions in pain after orgasm, potentially related to endorphin release and autonomic changes (Hämmerli et al., 2020). While not a replacement for medical treatment, these effects may contribute to subjective relief during rehabilitation (Hämmerli et al., 2020). Injury often leads to frustration, anxiety, or depressive symptoms (Dzieciatkowska et al., 2025). Emotional and sexual intimacy may help buffer these effects by providing comfort, connection, and stress relief, which indirectly supports recovery (Otadi, 2024). Depending on the type and severity of injury, certain movements or positions may be contraindicated (Medicine CfSC, 2010). Communication with medical professionals may help athletes maintain safe levels of physical activity, including sexual activity, during rehabilitation periods (Otadi, 2024).

### 3.5 Potential risks and negative outcomes

Although generally neutral or beneficial, sexual activity may have negative effects on recovery under specific conditions. The primary risk is sleep disruption, particularly when sexual activity occurs late at night and reduces total sleep duration or delays sleep onset (Chaput et al., 2020; Fernández-Lázaro et al., 2025b). Interpersonal stress, dissatisfaction, or conflict related to sexual relationships may also increase psychological stress and negatively affect recovery processes (Soller et al., 2017; Maïn-Ndeiang et al., 2025). In some cases, perceived fatigue immediately following sexual activity may be reported, although this is typically short-lived and context-dependent. Additionally, in athletes with certain musculoskeletal injuries, sexual activity may need to be modified to avoid pain or mechanical strain (Rosenbaum, 2010).

Sexual activity may contribute to recovery primarily through relaxation, emotional regulation, and potential improvements in sleep initiation. However, these effects are highly dependent on timing and psychological context. When appropriately timed and occurring in a supportive environment, sexual activity is unlikely to impair and may modestly support recovery processes. Negative effects are most consistently linked to sleep disruption and psychological stress rather than physiological mechanisms.

## 4 Physiological and psychological basis of sexual activity in athletes

Understanding the effects of sexual activity on athletic performance and recovery requires examining the underlying biological and psychological mechanisms triggered before, during, and after sexual encounters. Sexual activity involves a coordinated response across the endocrine, cardiovascular, neuromuscular, and autonomic systems (Carmichael et al., 1994). These responses may influence fatigue, energy availability, alertness, and mood, all of which are relevant to competitive performance (Ihalainen et al., 2024). Additionally, sexual activity is influenced by emotional and social dynamics that can support or hinder resilience, motivation, and overall readiness for sport (Soori et al., 2017; Fan et al., 2023). This section summarizes the main physiological and psychological pathways underlying sexual activity, performance, and recovery. **Table 2** providing a synthesized overview of the key hormonal, cardiovascular, neuromuscular, autonomic, and psychological responses and their potential athletic relevance, as supported by the evidence reviewed in the following subsections.

**Table 2:** Summary of physiological and psychological responses to sexual activity and their potential relevance to athletic performance and recovery.

System/response	Changes during/arousal	Changes after/post-orgasm	Potential athletic relevance
Hormonal	Testosterone ↑	Prolactin ↑, oxytocin ↑, cortisol ↓ (if relaxed)	May influence aggression and competitive drive during arousal; promotes relaxation, stress reduction, and mood stabilization post-orgasm.
Cardiovascular	HR ↑, BP ↑, metabolic cost low (mild exercise equivalent)	Parasympathetic activity ↑ (rebound), HR ↓, relaxation	Minimal energy expenditure; parasympathetic rebound may support post-exercise recovery.
Neuromuscular	Muscle tension ↑, pelvic floor activation	Muscle tension ↓	Transient tension during activity may affect readiness if timed too close to competition; post-orgasm relaxation may reduce pre-competition tightness.
Autonomic	Sympathetic ↑ (arousal, preparation)	Parasympathetic ↑ (recovery, relaxation)	Supports arousal regulation; parasympathetic dominance may improve sleep onset and reduce anxiety.
Psychological	Arousal ↑, focus variable	Mood ↑, bonding ↑, anxiety ↓ (in supportive contexts)	May enhance emotional readiness, focus, and motivation; supportive relationships can buffer stress.
Sleep	NA	Sleep onset latency ↓, perceived quality ↑ (if not late-night)	Improved sleep quality supports recovery and next-day performance when sexual activity is appropriately timed.

HR, heart rate; BP, blood pressure; not applicable.

↑ Indicates an increase.

↓ Indicates a decrease.

### 4.1 Hormonal responses

Sexual activity results in acute changes in several hormones that may influence performance. Testosterone typically increases during sexual arousal and may remain elevated for a short period afterward (O’Connor et al., 2004). As testosterone plays a role in muscle activation, aggression, and competitive drive, some have suggested that sexual activity could enhance performance under certain circumstances (O’Connor et al., 2004; Sgrò and Di Luigi, 2017). Conversely, other hormones such as prolactin rise immediately after orgasm, which is associated with reduced arousal, increased relaxation, and a transient dip in motivation or alertness (Kruger et al., 2003). Cortisol, a marker of physiological stress, may also fluctuate during and after sexual activity. Lower cortisol levels following sexual activity may promote relaxation and improved sleep, potentially supporting recovery (Hamilton et al., 2008; De Nys et al., 2022). However, excessive psychological stress linked to sexual concerns may raise cortisol and impair performance (Lautenbach et al., 2015; Omona and Ssanyu, 2025). These hormonal dynamics suggest that timing, context, and individual variability influence whether sexual activity has beneficial or neutral effects for athletes.

### 4.2 Cardiovascular, metabolic and autonomic responses

Sexual activity induces physiological changes that resemble mild to moderate physical exercise, including increases in heart rate, respiration rate, and blood pressure during sexual arousal and orgasm (Levin, 2007; Xue-Rui et al., 2008; Oliva-Lozano et al., 2022). The metabolic cost is relatively low, and the cardiovascular activation is generally insufficient to affect energy stores or performance (Soori et al., 2017; Oliva-Lozano et al., 2022). This acute sympathetic activation is followed by a shift toward parasympathetic dominance during the resolution phase, which may help with post-exercise relaxation, stress reduction, and sleep quality (Park et al., 2024). For some individuals, the temporary cardiovascular load is too small to affect performance, while for others, mild exertion close to competition might contribute to perceived fatigue (Kirecci et al., 2022; Zavorsky and Brooks, 2022). As with other physical stressors, the effects depend heavily on timing, individual fitness, and the nature of the activity (Zavorsky and Brooks, 2022). This autonomic balance has implications for sports where optimal arousal levels influence performance (Rowland and van Lankveld, 2019).

### 4.3 Neuromuscular and fatigue considerations

Neuromuscular responses during sexual activity include transient increases in muscle tension, contractions of pelvic and surrounding musculature, and varying degrees of physical exertion depending on duration and intensity (Faucher et al., 2024). These responses are generally mild when compared to sport-specific training (Giagio et al., 2021; Duarte et al., 2022). However, a minority of athletes may experience short-term muscular fatigue, particularly if sexual activity occurs shortly before competition (Soori et al., 2017). On the other hand, the relaxation that follows orgasm may lead to reduced muscular tension and improved readiness, especially for athletes who experience pre-competition anxiety or muscle tightness (Thornton, 1990). Whether these neuromuscular changes benefit or hinder performance is likely influenced by sport demands, individual arousal regulation, and personal psychological preferences.

### 4.4 Psychological and relational context

Sexual activity can significantly affect psychological states and occurs within interpersonal relationships, both of which directly influence athletic performance and recovery (SayfollahPour et al., 2013; Campbell et al., 2016). It may reduce anxiety, enhance mood, increase emotional bonding, and strengthen a sense of well-being (Mollaioli et al., 2021). These psychological benefits can contribute to improved focus, confidence, and resilience (SayfollahPour et al., 2013). Research in sport psychology consistently links positive mood and emotional stability to better training adherence and lower perceived exertion (Biscardi et al., 2024). The relational context is crucial. Supportive and healthy relationships provide emotional stability, improved coping with stress, and a sense of security (Peixoto and Sousa, 2023). These factors are associated with enhanced mental health and stronger recovery from injury (Rice et al., 2016; Otadi, 2024). Emotional intimacy and partner support may also buffer athletes against the pressures of competitive environments (Kent et al., 2025). In contrast, psychological stress related to sexual relationships, performance anxiety, interpersonal conflict, or cultural prohibitions can negatively impact focus, motivation, hormonal balance, and sleep (Troxel et al., 2007; Mbiydzenyuy et al., 2024; Otadi, 2024; Kent et al., 2025). Athletes under interpersonal strain may experience higher cortisol levels, poorer emotional regulation, and difficulties maintaining training focus (McLoughlin et al., 2021). Therefore, the psychological and social impact of sexual activity is highly individual and dependent on emotional context, relationship satisfaction, cultural beliefs, and personal expectations.

## 5 Behavioral, social, and lifestyle factors

Sexual activity in athletes does not occur in isolation but is shaped by behavioral habits, social environments, relationship dynamics, and the demands of athletic lifestyles (Miller et al., 1998). These factors influence not only the frequency and timing of sexual activity but also its psychological and physiological impact. Understanding these broader contexts is essential because they can moderate how sexual activity affects performance, recovery, and overall well-being. This section examines lifestyle routines, relationship factors, travel schedules, stress management, and risk behaviors that commonly interact with sexual behavior in athlete populations.

### 5.1 Travel, competition schedules, and stress

Travel is a major component of competitive sport and may influence sexual activity patterns as well as recovery quality (Heggie et al., 2013). Athletes traveling across time zones experience jet lag, altered routines, and disrupted sleep (Biggins et al., 2022). These factors often reduce sexual activity due to physical fatigue or scheduling conflicts. For some, however, sexual activity may help reduce stress or promote relaxation during travel-heavy periods (Bodenmann et al., 2010). Athletes vary in how they manage pre-competition anxiety. Some may avoid sexual activity due to concerns about distraction or fatigue, while others use it as a stress-reducing strategy. Emotional state significantly influences the perceived effect of sexual activity on readiness (Zimmer-Gembeck et al., 2015).

### 5.2 Relationship dynamics and emotional environment

Relationship quality is a key determinant of how sexual activity influences athlete well-being. Supportive relationships are associated with improved emotional stability, reduced stress, and better recovery behaviors, which may indirectly benefit training consistency and psychological readiness (Troxel et al., 2007; SayfollahPour et al., 2013; Mbiydzenyuy et al., 2024; Kent et al., 2025). In contrast, relationship conflict, dissatisfaction, or communication difficulties can elevate psychological stress and negatively affect sleep and recovery processes (Loving et al., 2004; Troxel et al., 2007; Stern et al., 2023). In these cases, the emotional context of sexual activity is more influential than the activity itself, reinforcing the importance of psychological environment in athlete recovery.

### 5.3 Social environment and team culture

Team environments and social norms influence attitudes toward sexual behavior. Athletes may experience pressure, expectations, or cultural messages that shape how they engage in sexual activity (Miller et al., 1998). Some sports cultures promote sexual abstinence before competition, while others are more permissive or neutral. Athletes often internalize these beliefs, which can create psychological expectations that influence perceived performance outcomes (Fischer, 1997; Alonso Aubin et al., 2023). Teammates can reinforce sexual norms, whether through encouragement, humor, or superstition (Graupensperger et al., 2018). These influences may affect an athlete’s decision making regardless of scientific evidence.

### 5.4 Risk-taking behavior in athlete populations

Athletes, particularly those at elite levels, may display sensation-seeking or risk-taking tendencies that extend to their sexual behavior. Nightlife, competition celebrations, and social events may contribute to late-night sexual encounters, alcohol use, or insufficient sleep, all of which can impair recovery and performance (Perkins, 2002; Devenney et al., 2019; Rodrigues et al., 2019). These behaviors pose greater risks than sexual activity itself. Media attention, fan interactions, and celebrity status may create pressure or complicate personal relationships (Kim and Song, 2016). Stress arising from public image concerns can influence sexual behavior and indirectly affect performance.

### 5.5 Personal beliefs and psychological preferences

Individual beliefs strongly influence how athletes regulate sexual activity around training and competition. Some athletes use sexual abstinence or sexual activity as part of personal rituals for emotional regulation or superstition (Alonso Aubin et al., 2023). These rituals can influence perceived readiness even when physiological effects are minimal. Athletes differ significantly in how sexual activity affects their arousal, aggression, and relaxation (Anshel, 1981). Personal comfort, confidence, and psychological patterns often determine whether sexual activity enhances or interferes with performance (Stoeber and Harvey, 2016).

Behavioral patterns, relationship quality, travel demands, and cultural beliefs shape how sexual activity affects athletes (Miller et al., 1998; Jowett and Cramer, 2009; Heggie et al., 2013; Rintaugu et al., 2020), with these psychological and contextual factors often exerting a greater influence on recovery and performance than physiological responses alone (Ayranci and Aydin, 2025). Understanding the broader lifestyle context allows athletes and practitioners to make individualized decisions rather than relying on rigid rules or traditional assumptions.

## 6 Sex differences

Sex differences play an important role in understanding how sexual activity influences athletic performance and recovery. Biological, hormonal, psychological, and social factors differ substantially between male and female athletes (Singh and Parmar, 2016; Hunter et al., 2023; Kuo et al., 2025), and these differences may shape how sexual activity affects strength, endurance, arousal, sleep, and psychological readiness. Research in this area is limited, and much of the available evidence is based on male samples. Therefore, interpretations must be approached with caution. This section summarizes known sex-specific differences and highlights areas requiring further study, with **Table 3** providing a synthesized overview of these differences based on evidence reviewed in the following subsections.

**Table 3:** Differences between male and female athletes in responses to sexual activity.

Aspect	Male athletes	Female athletes
Hormonal response	Testosterone: Rises during arousal; may remain elevated briefly post-orgasm. Prolactin: Increases post-orgasm, linked to relaxation/satiety. Cortisol: May decrease post-activity if relaxed.	Oxytocin: Often elevated post-orgasm, linked to bonding/relaxation. Cortisol: More variable; may decrease if stress is reduced. Estrogen/progesterone: Cycle-dependent; may influence arousal, mood, and recovery.
Cardiovascular and metabolic load	Slightly higher energy expenditure on average. Heart rate/BP increases similar to mild exercise. Quick return to baseline post-activity.	Energy expenditure generally lower but variable. Cardiovascular response may be more influenced by emotional state. Parasympathetic rebound may be more pronounced in some.
Fatigue and recovery perception	Minimal impact on next-day performance. Some report transient relaxation or reduced aggression immediately post-activity.	More variable: may enhance relaxation and sleep quality, or cause discomfort depending on cycle phase. Menstrual symptoms (cramps, fatigue) may interact with sexual activity and recovery.
Psychological and relational context	Often views sex as stress relief or energy release. Performance may be linked to confidence/aggression beliefs. Less societal scrutiny regarding sexual behavior.	Emotional connection and relationship satisfaction more strongly influence experience. May face greater societal stigma or privacy concerns. Sexual activity may be more mood- and recovery-sensitive.

BP, blood pressure.

### 6.1 Hormonal differences in males and females

In males, sexual activity is commonly associated with short-term increases in testosterone during arousal, followed by variable responses after orgasm (Fernández-Lázaro et al., 2025a). Testosterone is relevant to aggression, muscle activation, confidence, and competitive behavior (Estrada et al., 2003; Carré et al., 2010). Some theories suggest that abstinence may increase testosterone (McGlone and Shrier, 2000), but scientific findings are inconsistent, with many studies reporting minimal changes in baseline levels (Zavorsky and Brooks, 2022). In females, sexual activity may influence oxytocin and cortisol regulation, but effects vary depending on menstrual cycle phase, hormonal contraceptive use, and psychological context (Caruso et al., 2014). Unlike males, acute fluctuations in sex hormones after sexual activity may have less direct impact on muscle performance but may influence mood, relaxation, and sleep quality (Soori et al., 2017; Lastella et al., 2019; Zavorsky and Brooks, 2022).

### 6.2 Menstrual cycle considerations

Hormonal variations during the menstrual cycle affect mood, arousal, energy levels, thermoregulation, and recovery capacity (Caruso et al., 2014; Baker et al., 2020). During the follicular and ovulatory phases, elevated estrogen may enhance muscle function, endurance, and motivation (Elorduy-Terrado et al., 2025). Although research on sexual activity and menstrual cycle phase remains limited and somewhat variable, several studies suggest that sexual desire and activity may be more frequently reported during the late follicular and peri-ovulatory phase (Bullivant et al., 2004; Kiesner et al., 2025). Higher progesterone levels during the luteal phase may increase fatigue, disrupt sleep, and influence mood (Ziomkiewicz et al., 2012; Cherpak and Van Lare, 2020). Sexual activity may improve relaxation for some females during this phase, while others may experience discomfort or reduced libido, influencing participation (Clayton et al., 1999). Menstrual pain, cramping, or fatigue may affect willingness to engage in sexual activity and may also interact with recovery processes (Guillermo et al., 2010). Some females report improved mood or pain relief from sexual activity during menstruation, while others experience increased discomfort (Fahs, 2020).

### 6.3 Differences in physiological load and fatigue

Males generally experience slightly higher energy expenditure and cardiovascular activation during sexual activity (Frappier et al., 2013). For most, this exertion is too low to affect performance (Zavorsky et al., 2019; Zavorsky and Brooks, 2022), but immediately timed activity may contribute to mild fatigue in a small subset of men (Bohlen et al., 1984). Physiological responses during sexual activity tend to be more variable in women (Stanton et al., 2015). Some experience prolonged relaxation and improved parasympathetic activation, which may support sleep and recovery. Others may show a smaller autonomic shift, leading to fewer noticeable physical effects (Stanton et al., 2015).

### 6.4 Psychological and behavioral differences

Women often report greater emotional and relational influences on sexual activity (Kokka et al., 2025). Relationship satisfaction, stress levels, and emotional connection may more strongly affect females’ experiences and outcomes (Davison et al., 2009). When sexual activity enhances emotional well-being, recovery may benefit. When linked to conflict or stress, recovery may decline (Ein-Dor and Hirschberger, 2012; Mollaioli et al., 2021). Males may place more emphasis on sexual activity as a method of relaxation or energy release, although substantial individual variation exists (Bancroft et al., 2003). Females may experience greater societal scrutiny regarding sexual behavior, which can influence stress, privacy concerns, and psychological responses (Crawford and Popp, 2003). Males often face more permissive social norms, but may also encounter pressure to conform to stereotypes involving aggression or masculinity (Connell and Messerschmidt, 2005; Potts, 2014; Wong et al., 2017).

### 6.5 Sport-specific considerations

The physiological and psychological demands of sport vary widely across disciplines, suggesting that the effects of sexual activity may not be uniform. In strength and power sports such as weightlifting and sprinting, where performance relies on high levels of neuromuscular activation and aggression, acute hormonal fluctuations, particularly in testosterone, could theoretically influence readiness (Cardinale and Stone, 2006; Carré et al., 2010). Conversely, in endurance sports such as marathon running and cycling, factors such as recovery, sleep quality, and autonomic balance may be more relevant (Ramos-Campo et al., 2019; Reichel et al., 2022). Technical or precision-based sports such as archery and shooting depend heavily on fine motor control, focus, and emotional regulation (Wu et al., 2021; Lulu et al., 2025), where the psychological aftermath of sexual activity, such as reduced anxiety or, alternatively, distraction, could play a decisive role. Combat sports present a unique intersection of aggression, weight management, and pre-competition arousal regulation (Pettersson et al., 2013), where beliefs about sexual abstinence are often deeply entrenched (Vajda and Reguli, 2018). Emerging evidence confirms sport-specific hormonal responses in female athletes: soccer increases both testosterone and cortisol; netball elevates cortisol while reducing the testosterone-to-cortisol ratio; volleyball increases testosterone; and handball raises cortisol without changing testosterone (Khaleghi and Ahmadi, 2025). Despite these nuances, few studies have systematically compared outcomes across disciplines, and even fewer in female athletes, limiting sport-specific guidance.

### 6.6 Research gaps in sex differences

The majority of studies on sexual activity and performance are conducted in male populations, leaving major gaps in female-focused research.

Key limitations include:

- Lack of controlled studies in female athletes.
- Limited understanding of interaction between sexual activity and menstrual cycle.
- Insufficient data on hormonal contraceptive influences.
- Scarcity of sport-specific analyses for women.
- Underrepresentation of female athletes in psychophysiological studies.

More balanced research is needed to develop sex-specific guidelines and to understand the unique biological and psychological mechanisms in women.

Sex differences significantly shape the physiological and psychological effects of sexual activity on athletes. While male athletes show more predictable hormonal responses, female athletes exhibit greater variability due to menstrual cycle phases, emotional context, and social influences. The current evidence base is limited, particularly for women, highlighting the need for future research that incorporates sex-specific factors to better inform individualized guidance for athletes.

## 7 Clinical and practical implications

Understanding the relationship between sexual activity, athletic performance, and recovery has meaningful implications for athletes, coaches, sports physicians, and psychologists. Although research findings indicate that sexual activity rarely has a direct negative impact on physical performance (Stefani et al., 2016; Soori et al., 2017; Zavorsky and Brooks, 2022), individual differences, timing considerations, lifestyle factors, and psychological responses can significantly influence outcomes. This section outlines practical considerations for applying scientific knowledge in real-world athletic settings and emphasizes the importance of individualized guidance rather than rigid one-size-fits-all recommendations. **Table 4** summarizes key practical guidelines.

**Table 4:** Practical guidelines for athletes managing sexual activity, performance, and recovery.

Area of focus	Recommendation	Rationale/evidence summary
Timing before competition	Avoid sexual activity immediately before competition if it leads to relaxation, reduced arousal, or perceived fatigue.	Evidence shows no impairment in performance when sex occurs the night before. Immediate pre-event timing may affect psychological readiness.
Sleep and recovery	Prioritize sleep hygiene: avoid late-night sexual activity during heavy training cycles, especially if early morning training follows. Earlier-evening timing may support sleep onset without reducing total sleep duration.	Late-night activity can delay sleep onset and reduce sleep duration, impairing recovery. Sexual activity earlier in the evening may improve sleep quality via parasympathetic rebound.
Individual monitoring	Encourage athletes to self-monitor responses (energy, mood, sleep, performance) using journals or apps to identify personal patterns.	Responses are highly individual. Self-awareness helps tailor timing and frequency to personal recovery and performance needs.
Relationship context	Foster open communication with partners about training schedules, recovery needs, and emotional support. Address conflicts proactively to avoid stress that may impair recovery.	Supportive relationships enhance emotional stability and recovery. Conflict or tension can elevate cortisol and disrupt sleep.
Psychological readiness	Use sexual activity as a relaxation tool if it reduces pre-competition anxiety. Avoid if it induces guilt, distraction, or stress.	Psychological state often outweighs physiological effects. Positive emotional context can enhance focus and resilience.
Injury and medical considerations	Consult a sports physician if sexual activity causes pain, discomfort, or concerns related to injury. Modify positions/activity as needed.	Certain injuries may be aggravated by sexual activity. Medical guidance ensures safety and supports continued intimacy during rehabilitation.
Cultural and personal beliefs	Respect personal and cultural beliefs regarding abstinence or activity. Avoid imposing universal rules; support athlete autonomy in decision-making.	Beliefs strongly influence perceived readiness. Forced abstinence or activity may cause psychological stress that impairs performance.

### 7.1 Individualized decision making for athletes

Athletes vary widely in how sexual activity affects mood, arousal, energy levels, motivation, and sleep (Sgrò and Di Luigi, 2017; Rowland and van Lankveld, 2019). Because these responses differ across individuals, personalized approaches are necessary. Encouraging athletes to observe how sexual activity affects their own performance, sleep, and psychological state can help them make informed decisions (SayfollahPour et al., 2013; Lastella et al., 2019; Zavorsky and Brooks, 2022). Journaling or subjective monitoring tools may help athletes identify patterns and adjust behavior according to training demands (Nässi et al., 2017). Some athletes feel more relaxed and focused after sexual activity, while others prefer abstinence due to personal beliefs or psychological preference (Anshel, 1981; Vajda and Reguli, 2018; Alonso Aubin et al., 2023; Otadi, 2024). Reinforcing that both responses are valid allows athletes to adopt strategies that align with their own comfort and performance needs.

### 7.2 Timing of sexual activity relative to training and competition

As reviewed in earlier sections, sexual activity is unlikely to impair performance if it occurs several hours or the night before competition (Stefani et al., 2016; Soori et al., 2017; Zavorsky and Brooks, 2022). However, activity immediately before may cause relaxation or fatigue (Levin, 2007; Sgrò and Di Luigi, 2017). Recommendations should therefore emphasize timing rather than strict abstinence. During heavy training periods, athletes should prioritize sleep quality and recovery routines (Bird, 2013; O’Donnell et al., 2018). Sexual activity late at night may reduce sleep duration (Chaput et al., 2020), especially when training sessions begin early in the morning. Earlier-evening sexual activity may minimize this effect. After competition, sexual activity may support relaxation and emotional recovery (Anshel, 1981; Sgrò and Di Luigi, 2017). Athletes who experience difficulty winding down after competition may benefit from the calming effects associated with sexual activity.

### 7.3 Psychological considerations in performance planning

Psychological responses to sexual activity, including mood improvement, reduced anxiety, or emotional support, can influence performance and recovery (Zimmer-Gembeck et al., 2015; Otadi, 2024). For athletes who struggle with performance anxiety, sexual activity may serve as a psychological relaxation method. However, if sexual activity is associated with stress, guilt, or interpersonal conflict, it may worsen anxiety (Brotto et al., 2016). Stable, supportive relationships can contribute to emotional well-being, which plays an important role in decision making, focus, and motivation (Reis, 2001). Coaches and sports psychologists should recognize the influence of emotional intimacy on athlete mental health.

### 7.4 Clinical guidance from sports physicians

Sports physicians may provide guidance when athletes have concerns about injury, pain, or medical conditions that influence sexual activity. Certain injuries, particularly those involving the lower back may require modified sexual activity to avoid discomfort or strain (Rosenbaum, 2010). Physicians can provide safe guidelines based on injury type and stage of healing. Physicians can help athletes understand how sexual activity interacts with sleep patterns or hormonal fluctuations, especially in female athletes who experience symptoms related to menstrual cycles. Medical professionals can correct myths about testosterone depletion, energy loss, or performance deficits that lack scientific support.

### 7.5 Recommendations for coaches and support staff

Coaches should approach discussions about sexual activity with sensitivity, professionalism, and respect for privacy. Universal bans on sexual activity before competition are not supported by evidence (Stefani et al., 2016; Soori et al., 2017; Zavorsky and Brooks, 2022). Coaches should avoid imposing restrictive rules unless there are clear performance or behavioral concerns. Athletes benefit from autonomy in managing personal decisions, including sexual activity. Coaches can encourage open communication with medical or psychological staff if concerns arise. Coaches should be aware that risky behaviors associated with nightlife, alcohol use, or late-night encounters may impair performance far more than sexual activity itself.

### 7.6 Practical guidelines for athletes

Based on the current evidence, the following general guidelines may help athletes manage sexual activity in harmony with performance and recovery goals:

1. Monitor personal responses to sexual activity and adjust behavior accordingly.
2. Avoid sexual activity immediately before competition if it leads to relaxation or reduced focus.
3. Prioritize sleep by avoiding very late-night sexual activity during heavy training periods.
4. Engage in open communication with partners about training schedules and recovery needs.
5. Address relationship conflict or emotional stress that may undermine performance.
6. Consult medical professionals when injuries or hormonal concerns arise.

Sexual activity has diverse effects on athletes, shaped by timing, psychological context, lifestyle factors, and individual variability. Clinicians and coaches should take a balanced, evidence-informed approach that respects athlete autonomy and emphasizes personal awareness. Rather than endorsing rigid abstinence rules, practical guidance should focus on optimizing timing, supporting emotional well-being, and maintaining healthy recovery routines.

## 8 Gaps in the literature and future directions

Although interest in the relationship between sexual activity, athletic performance, and recovery has increased, the current evidence base remains limited by methodological weaknesses, small sample sizes, and inconsistent study designs (Zavorsky and Brooks, 2022). There is a lack of high-quality experimental research that uses standardized protocols, objective physiological measurements, or systematic assessment of timing effects (Zavorsky and Brooks, 2022). Future studies should incorporate controlled laboratory designs, randomized or counterbalanced procedures, and precise physiological monitoring such as hormonal assays, heart rate variability, and sleep tracking in order to establish clearer causal relationships. A major gap in the literature is the underrepresentation of female athletes (Stefani et al., 2016; Soori et al., 2017; Zavorsky and Brooks, 2022). Most existing studies involve male participants, leaving significant unanswered questions about how sexual activity interacts with menstrual cycle phases, hormonal contraceptive use, and female-specific physiological or psychological responses. Understanding these factors is essential for developing sex-specific and evidence-based recommendations. Additionally, very few investigations consider sport-specific differences. Athletes in strength sports, endurance sports, combat sports, team sports, and aesthetic or skill-based disciplines experience unique physical and psychological demands, yet research rarely distinguishes outcomes among these groups (Soori et al., 2017). Clarifying how sexual activity affects performance across different sport categories would enhance the relevance of findings for practitioners. Studying sexual activity also presents methodological challenges due to privacy concerns, social desirability bias, and the wide variability in sexual behaviors (Fenton et al., 2001). More accurate and confidential reporting methods, clearer operational definitions of sexual activity, and standardized timing categories are needed. Integrating wearable technology (e.g., heart rate variability monitors, sleep trackers) can improve data quality by providing objective, continuous measurements of autonomic recovery, sleep onset latency, and sleep stage distribution. When combined with event logging, wearables reduce recall bias, increase temporal precision, and clarify whether late−night sexual activity meaningfully affects restorative sleep and next−day performance. Privacy and data security concerns must, however, be addressed when collecting such intimate physiological data from athletes.

Recovery-related outcomes represent another understudied area. While sexual activity may influence sleep, hormonal regulation, muscle repair, and emotional restoration, few studies directly measure these recovery markers (SayfollahPour et al., 2013; Stefani et al., 2016; Soori et al., 2017; Zavorsky and Brooks, 2022). Future research should examine sleep architecture, inflammatory biomarkers, hormonal responses, muscle soreness indicators, and psychological resilience following sexual activity. Cultural and psychological influences also remain poorly understood (Miller et al., 1998). Beliefs about abstinence, relationship satisfaction, emotional support, and cultural or religious norms can shape how athletes perceive the effects of sexual activity and may exert stronger influence than biological factors (Miller et al., 1998). Cross-cultural studies and research integrating sport psychology perspectives are needed to understand these contextual variables. Finally, the field would benefit from longitudinal research that tracks athletes over training cycles or competitive seasons to determine long-term patterns. Real-world monitoring of sleep, mood, sexual behavior, training load, menstrual cycle (for female athletes), and performance would provide more applicable insights for daily practice.

Overall, significant gaps remain in the scientific understanding of how sexual activity influences performance and recovery. Addressing these limitations through larger sample sizes, sex-balanced designs, rigorous experimental methods, and interdisciplinary collaboration will help produce stronger evidence and support more precise, individualized recommendations for athletes and support staff.

## 9 Conclusion

Sexual activity has a largely neutral effect on athletic performance when appropriately timed. Physiological responses are mild and transient, while psychological and contextual factors, particularly sleep, timing, and individual perception, play a more important role in determining outcomes. Evidence does not support universal abstinence before competition. Instead, effects vary between individuals and are influenced more by behavioral and psychological context than by direct physiological mechanisms. In some cases, sexual activity may support relaxation and emotional well-being, while in others it may be neutral or slightly disruptive if poorly timed. Overall, sexual activity should be understood as part of an athlete’s broader lifestyle rather than a performance-limiting factor. Individualized strategies based on personal response, sleep quality, and training demands are most appropriate.
